# Prevalence of somatic diseases in adults with attention deficit hyperactivity disorder in Japan is highest in people aged ≥40 years with mental disorders: a cross-sectional study of a Japanese health insurance claims database

**DOI:** 10.3389/fpsyt.2024.1197513

**Published:** 2024-02-14

**Authors:** Yoshikazu Takaesu, Yumi Sato, Shinpei Iwata, Patcharapim Takizawa, Hideyuki Miyauchi, Yoshikazu Ishimoto, Tsuyoshi Kondo

**Affiliations:** ^1^ Department of Neuropsychiatry, Graduate School of Medicine, University of the Ryukyus, Okinawa, Japan; ^2^ Medical Affairs Department, Integrated Disease Care Division, Shionogi & Co., Ltd., Osaka, Japan; ^3^ PMS & Pharmacoepidemiology Department, Shionogi Pharmacovigilance Center Co., Ltd., Osaka, Japan; ^4^ Japan Medical Office, Takeda Pharmaceutical Co., Ltd., Tokyo, Japan

**Keywords:** attention deficit hyperactivity disorder, health insurance claims database study, observational, chronic diseases, somatic diseases, stratified analysis, mental disorders, Japan

## Abstract

**Introduction:**

Studies have reported an association between attention deficit hyperactivity disorder (ADHD) and somatic diseases; however, the correlation of mental disorders with the association between ADHD and somatic diseases remains uninvestigated. This study investigated and compared the prevalence of somatic diseases among adults with/without ADHD, stratified by the presence or absence of mental disorders.

**Methods:**

This cross-sectional study (October 2020–September 2021), using data (June 2013–September 2021) from a Japanese health insurance claims database, included adult participants with a medical record of and receiving medication for ADHD (ADHD group); the control group (matched 1:5 by age/sex) comprised participants without ADHD. The prevalence and odds ratio (OR; ADHD versus control) of type 2 diabetes mellitus (T2DM), diabetes complications, hypertension, cardiovascular disease (CVD), dyslipidemia, gout and hyperuricemia, chronic obstructive pulmonary disease (COPD), non-alcoholic fatty liver disease/non-alcoholic steatohepatitis (NAFLD/NASH), and atopic dermatitis were investigated. Pooled ORs for stratified analysis were calculated using the Mantel-Haenszel method.

**Results:**

In the matched analysis sets, the ORs for all somatic diseases were significantly higher for the ADHD group (n=15,028) versus the control group (n=74,796). On stratified analysis, the Mantel-Haenszel ORs were significant for NAFLD/NASH (1.53; 95% confidence interval [CI]: 1.34, 1.73), diabetes complications (1.39; 95% CI: 1.09, 1.77), and gout and hyperuricemia (1.34; 95% CI: 1.19, 1.51). Furthermore, the stratum-specific ORs for T2DM, hypertension, and dyslipidemia were >1 and <1 in the presence and absence of mental disorders, respectively. The prevalence of all somatic diseases except atopic dermatitis increased with age. For participants aged ≥40 years, the Mantel-Haenszel ORs were significant for all somatic diseases except CVD, COPD, and atopic dermatitis.

**Conclusions:**

The prevalence of several somatic diseases, including chronic disorders, was high among adults with ADHD, particularly in those aged ≥40 years and those with mental disorders.

## 1 Introduction

Attention deficit hyperactivity disorder (ADHD) is characterized by symptoms of inattention, hyperactivity, and impulsivity (1). It commonly co-exists with other conditions (2). The Japan National Health and Wellness Survey 2016 identified a significantly higher rate of physical and mental comorbidities among undiagnosed adults with potential ADHD symptoms versus those without symptoms (3). According to the World Federation of ADHD International Consensus Statement, the global occurrence of ADHD is 5.9% among youth and 2.5% among adults (4). In Japan, the prevalence of adult ADHD was reported as 2.0%–2.5% (5), and the annual incidence of adult ADHD was 6 per 10,000 person-years as reported by the National Database of Health Insurance Claims and Specific Health Checkups in 2018 (6).

Results from the National Comorbidity Survey Replication undertaken in the United States of America (US) showed an increased prevalence of a wide range of other mental disorders according to the Diagnostic and Statistical Manual of Mental Disorders (DSM)-4 in adults with ADHD (7). Similarly, the prevalence of psychiatric comorbidities was high among Japanese adults with ADHD in a post-marketing surveillance (PMS) study undertaken in Japan (8). This PMS study found that over 50% of adult patients with ADHD reported at least one co-existing psychiatric disorder, and of these, almost 40% reported multiple psychiatric disorders (8). Moreover, an analysis of data from the Adult Psychiatric Morbidity Survey conducted in the US in 2007 reported an increased risk of non-psychiatric comorbidities among patients with ADHD. According to this survey, the odds for possible ADHD were over three times higher in individuals who had ≥5 physical diseases than in those who had no physical disease (odds ratio [OR]: 3.30, 95% confidence interval [CI]: 2.48, 4.37) (9).

Several studies have identified a possible link between ADHD and non-psychiatric disorders (10), including cardiovascular disease (CVD) (11), atopic dermatitis (12, 13), and gout (14). The health-risk behavior exhibited by patients as a result of ADHD-associated symptoms and deficits along with a common underlying pathophysiology (15) may increase the risk of comorbidities, such as diabetes mellitus (4, 16–18), dyslipidemia (11), and hyperuricemia (14), in patients with ADHD. Moreover, a strong association between ADHD and nervous system disorders and respiratory, musculoskeletal, and metabolic diseases has been reported in a Swedish registry study (10). Furthermore, studies have also conducted multivariate analysis, adjusting for psychiatric comorbidities as confounding factors, to assess whether ADHD confers additional risk of non-psychiatric disorders, such as metabolic syndrome (19) and CVD (11). However, it has not been investigated how mental disorders correlate with the association between ADHD and somatic diseases, stratified by the presence or absence of mental disorders.

Using a large Japanese health insurance claims database, we conducted a cross-sectional study to investigate and compare the prevalence of somatic diseases among adults with ADHD and those without ADHD. Furthermore, using stratified analysis, we investigated the correlation of the presence or absence of mental disorders with the association between ADHD and somatic diseases.

## 2 Methods

### 2.1 Study design

**Figure 1:**
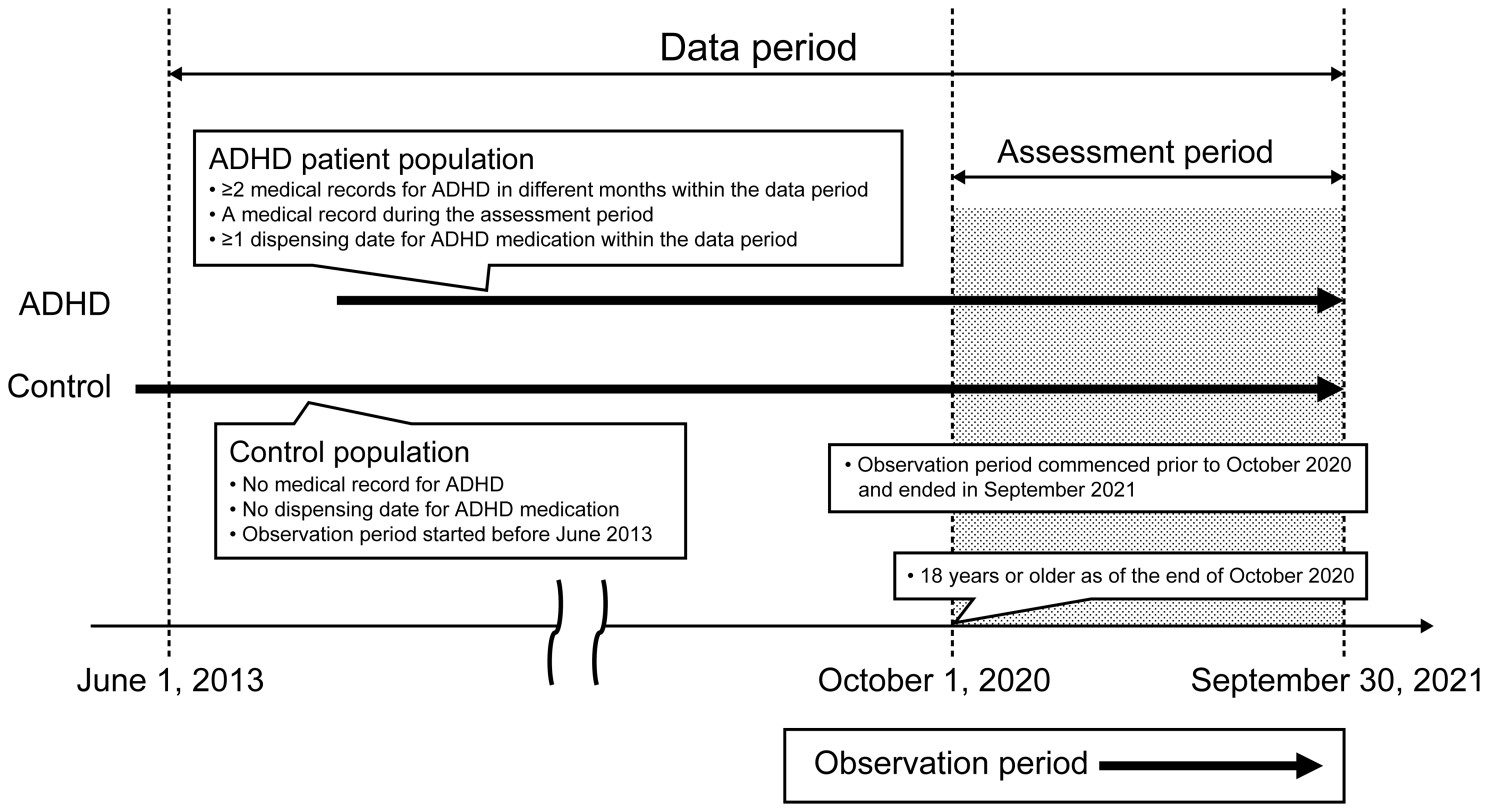
Study design. ADHD, attention deficit hyperactivity disorder.

This cross-sectional study was conducted using data extracted from the health insurance claims database developed by JMDC Inc. (Tokyo, Japan). Data from June 1, 2013, to September 30, 2021 (data period), were retrospectively collected from the database and assessed from October 1, 2020, to September 30, 2021 (assessment period) (**Figure 1**). The data period was chosen considering the approval date (August 24, 2012) for atomoxetine, the earliest approved drug for adults with ADHD in Japan, and the release date (May 2013) of DSM-5, a diagnostic criterion for ADHD.

This study was conducted in compliance with the Ethical Guidelines for Medical and Health Research Involving Human Subjects and its Guidance document (20). Informed consent was not required because the JMDC database contains Anonymized Processed Information, as defined in Article 2, Paragraph 6 of the Act on the Protection of Personal Information (Act No. 57 of 2003) (21). This study (UMIN000049401) is registered at the UMIN Clinical Trials Registry (https://www.umin.ac.jp/ctr/).

### 2.2 Medical information database

The JMDC claims database (hereafter, JMDC database) is a Japanese health insurance claims database that has been receiving medical information from multiple health insurance associations since 2005. As of September 2021, the JMDC database contained cumulative data from approximately 14 million enrollees, including employees and their dependents only. The JMDC database facilitates the tracking of medical information across multiple healthcare organizations for enrolled individuals unless they leave health insurance. The JMDC database comprises very few enrollees aged >65 years and no enrollees aged >75 years (22). This is because most enrollees in Japan exit the health insurance system on retirement at the age of 65 years, and the remaining enrollees aged >65 years exit by the age of 75 years.

### 2.3 Study population

This study included individuals aged ≥18 years (as of the end of October 2020). The observation period (the period for which an individual has been enrolled in/covered by health insurance) for a study participant began prior to October 2020 and ended in September 2021. The ADHD group included patients with at least two medical records for ADHD (International Classification of Diseases [ICD]-10 code: F90; no suspicion flag) in different months and at least one dispensing date for ADHD medication (methylphenidate [Anatomical Therapeutic Chemical {ATC} code: N06BA04]/atomoxetine hydrochloride [ATC code: N06BA09]/guanfacine hydrochloride [ATC code: C02AC02]) within the data period and a medical record for ADHD during the assessment period. The control group included individuals who had neither a medical record for ADHD nor a dispensing date for ADHD medication throughout the data period, and the observation period started before June 2013 (**Figure 1**).

Data were summarized for age (as of the end of October 2020), age group (18–29 years, 30–39 years, and ≥40 years), sex, initial year of ADHD diagnosis (ADHD group only; June–December 2013, 2014, 2015, 2016, 2017, 2018, 2019, 2020; January–September 2021), start year of observation period (<2013, 2014, 2015, 2016, 2017, 2018, 2019, 2020), and the presence or absence of mental disorders. The presence of mental disorders was defined as having a medical record for substance use disorder (ICD-10 codes: F10–F19; no suspicion flag), depression (ICD-10 codes: F32, F33; no suspicion flag), bipolar disorder (ICD-10 codes: F30, F31; no suspicion flag), or anxiety (ICD-10 codes: F40, F41, F42, F44, F45, F48; no suspicion flag) in a month during the data period (15).

### 2.4 Study outcomes

The comorbidities of interest identified in the ADHD and control groups using outcome definitions from previous database studies included type 2 diabetes mellitus (T2DM), diabetes complications, hypertension, CVD, dyslipidemia, gout and hyperuricemia, chronic obstructive pulmonary disease (COPD), non-alcoholic fatty liver disease/non-alcoholic steatohepatitis (NAFLD/NASH), and atopic dermatitis (**Supplementary Table 1**).

### 2.5 Statistical analysis

The ADHD and control groups comprising the analysis sets were matched by coarsened exact matching algorithm at a ratio of 1:5 based on age and sex.

The prevalence of outcomes in the ADHD and control populations was determined for the pre-matched study population and the matched analysis set; ORs of the ADHD population to the control population and 95% CIs were calculated for the matched analysis set. Stratified analyses were conducted using the presence or absence of mental disorders as stratification factors in the matched analysis set. For matched pairs, the observation period of the ADHD patient was shared with the control in order to align the period for defining the presence or absence of mental disorders. Pooled ORs and 95% CIs for the strata were calculated using the Mantel-Haenszel method. The Breslow-Day test was conducted to ascertain whether the magnitude of the effect of ADHD on the prevalence of a somatic disease differed between the strata (presence or absence of mental disorders). The *p* values for homogeneity between the strata were calculated. The Mantel-Haenszel OR could be interpreted only when homogeneity was not rejected by the Breslow-Day test; rejected when *p*<0.05. A *p* value of <0.05 on the Breslow-Day test indicated that the ORs were not homogeneous between the strata with and without mental disorders, rendering the Mantel-Haenszel OR meaningless. Therefore, for somatic diseases with *p*<0.05, Mantel-Haenszel ORs were not calculated. To complement the results of the stratified analyses, logistic regression analyses were also performed, in which the population (ADHD or control), the mental disorders (presence or absence), the mental disorders-age group, and the mental disorders-sex were set as explanation variables (the mental disorders-age group and the mental disorders-sex were interaction terms). Subgroup analyses by background factors, age categories (18–29 years, 30–39 years, and ≥40 years), and sex (male and female) were performed as secondary analyses. For the purpose of improving the robustness of the results, sensitivity analyses were performed, in which two types of criteria were applied to the definition of the ADHD population: (1) having a yearly medical record for ADHD and (2) having yearly medical records for both ADHD and dispensing dates for ADHD medication.

Statistical analyses were performed using SAS version 9.4 with a two-sided significance level of 5%.

## 3 Results

### 3.1 Study population and characteristics

**Figure 2:**
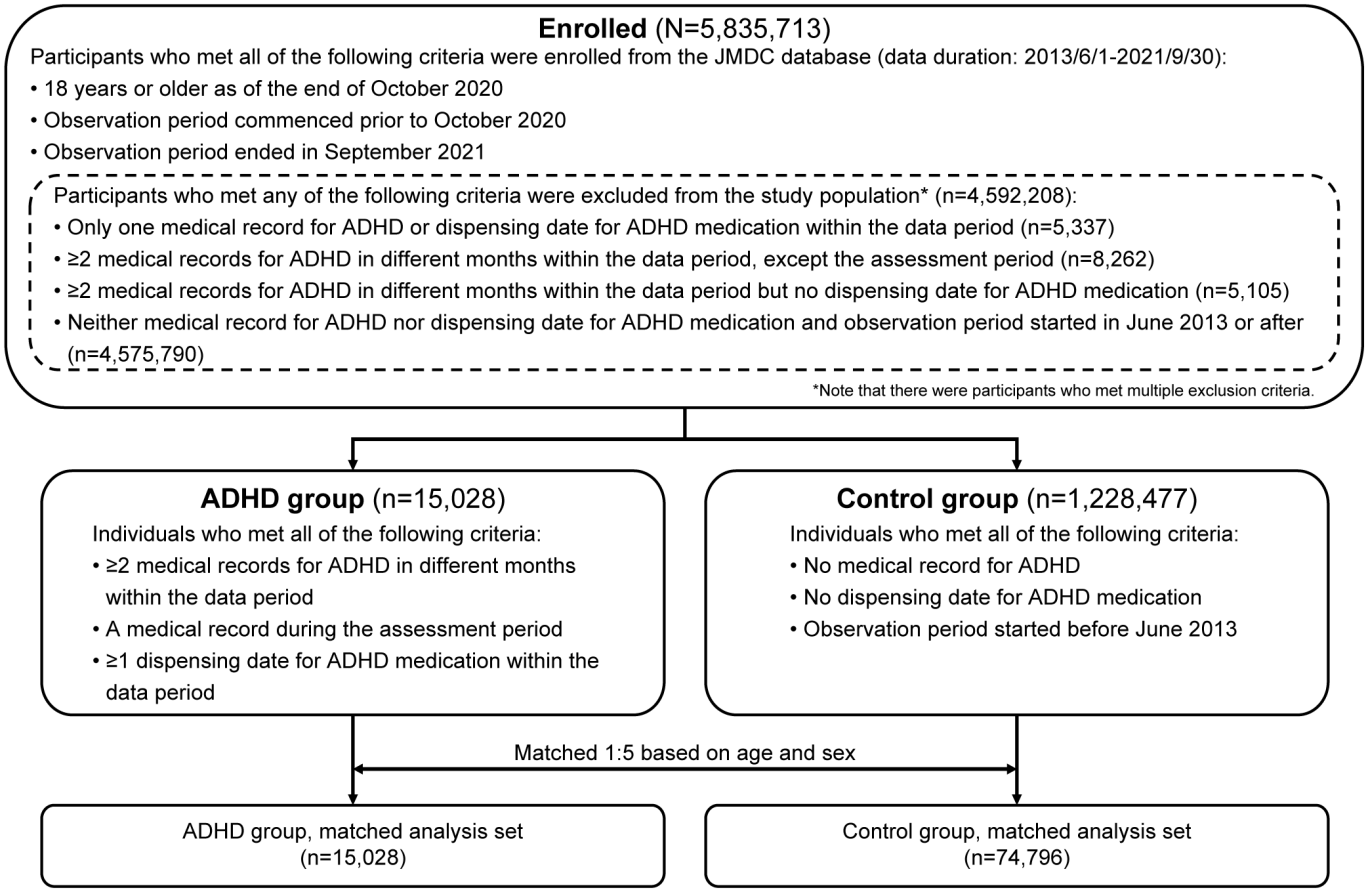
Participant flowchart. ADHD, attention deficit hyperactivity disorder.

Of the 5,835,713 participants aged ≥18 years enrolled in the JMDC database who met the inclusion criteria, 15,028 were included in the ADHD group and 1,228,477 in the control group (**Figure 2**). Furthermore, the ADHD and control groups were matched in a 1:5 ratio and included 74,796 participants in the control group for analysis. The ADHD group predominantly comprised men (60.7%; **Table 1**). The proportion of participants with ADHD showed a steep rise from 2017 onward (2014: 1.4%; 2017: 11.1%; and 2020: 25.4%). The proportion of patients in the start year of observation period in the ADHD group was almost evenly distributed (7.6% to 14.3%) except for 2014 (1.8%).

**Table 1:** Population demographics and characteristics.

		Pre-matched	Matched^*^
Variables	ADHD N=15,028 n (%)	Control N=1,228,477 n (%)	Control N=74,796 n (%)
Age, years			
**Mean ± SD**	33.1 ± 11.4	45.5 ± 12.8	33.2 ± 11.4
** 18–29 years**	6,912 (46.0)	150,672 (12.3)	34,216 (45.7)
** 30–39 years**	3,690 (24.6)	211,851 (17.2)	18,450 (24.7)
** ≥40 years**	4,426 (29.5)	865,954 (70.5)	22,130 (29.6)
Sex			
** Female**	5,903 (39.3)	463,323 (37.7)	29,171 (39.0)
** Male**	9,125 (60.7)	765,154 (62.3)	45,625 (61.0)
Initial year of ADHD diagnosis			
** 2013 (June–December)**	435 (2.9)	–	–
** 2014**	203 (1.4)	–	–
** 2015**	671 (4.5)	–	–
** 2016**	1,039 (6.9)	–	–
** 2017**	1,674 (11.1)	–	–
** 2018**	2,215 (14.7)	–	–
** 2019**	2,899 (19.3)	–	–
** 2020**	3,815 (25.4)	–	–
** 2021 (January–September)**	2,077 (13.8)	–	–
Start year of observation period			
** <2013**	1,599 (10.6)	760,601 (61.9)	44,008 (58.8)
** 2013**	1,136 (7.6)	467,876 (38.1)	30,788 (41.2)
** 2014**	278 (1.8)	0 (0)	0 (0)
** 2015**	1,917 (12.8)	0 (0)	0 (0)
** 2016**	1,841 (12.3)	0 (0)	0 (0)
** 2017**	2,096 (13.9)	0 (0)	0 (0)
** 2018**	2,110 (14.0)	0 (0)	0 (0)
** 2019**	2,148 (14.3)	0 (0)	0 (0)
** 2020**	1,903 (12.7)	0 (0)	0 (0)
Number of mental disorders			
** 0**	2,408 (16.0)	992,049 (80.8)	64,960 (86.8)
** ≥1**	12,620 (84.0)	236,428 (19.2)	9,836 (13.2)

^*^Population matched 1:5 for age and sex.

ADHD, attention deficit hyperactivity disorder; SD, standard deviation.

The proportion of participants with at least one mental disorder was higher in the ADHD group (84.0%) than in the control group (13.2%; **Table 1**). Furthermore, this proportion tended to increase with age in the ADHD group, was the highest (91.3%) in the ≥40-years age category (**Supplementary Table 2**), and was also higher in women (89.3%) than in men (80.6%) with ADHD (**Supplementary Table 3**).

### 3.2 Somatic diseases

In the matched analysis set, the ORs for all somatic diseases of interest for the ADHD group versus the control group were significantly higher (**Table 2**). The ORs for the ADHD group versus the control group for T2DM, dyslipidemia, COPD, and NAFLD/NASH were larger than two, and that for NAFLD/NASH was the highest (2.35; 95% CI: 2.14, 2.59; **Table 2**).

**Table 2:** Proportion of patients with somatic diseases.

		Pre-matched	Matched^*^	
Somatic diseases n (%)	ADHD N=15,028 n (%)	Control N=1,228,477 n (%)	Control N=74,796 n (%)	Odds ratio^**^ (95% CI)
**T2DM**	435 (2.9)	51,786 (4.2)	1,072 (1.4)	**2.05 (1.83, 2.29)**
**Diabetes complications**	155 (1.0)	17,988 (1.5)	400 (0.5)	**1.94 (1.61, 2.34)**
**Hypertension**	994 (6.6)	157,327 (12.8)	2,891 (3.9)	**1.76 (1.64, 1.90)**
**CVD**	448 (3.0)	56,378 (4.6)	1,256 (1.7)	**1.80 (1.61, 2.01)**
**Dyslipidemia**	1,104 (7.4)	136,009 (11.1)	2,722 (3.6)	**2.10 (1.95, 2.26)**
**Gout and hyperuricemia**	667 (4.4)	75,583 (6.2)	1,936 (2.6)	**1.75 (1.60, 1.91)**
**COPD**	251 (1.7)	16,208 (1.3)	576 (0.8)	**2.19 (1.89, 2.54)**
**NAFLD/NASH**	619 (4.1)	41,987 (3.4)	1,341 (1.8)	**2.35 (2.14, 2.59)**
**Atopic dermatitis**	1,331 (8.9)	53,110 (4.3)	4,432 (5.9)	**1.54 (1.45, 1.64)**

^*^Population matched 1:5 for age and sex.

^**^ Odds ratio for the ADHD/control group in the matched analysis set.

ADHD, attention deficit hyperactivity disorder; CI, confidence interval; COPD, chronic obstructive pulmonary disease; CVD, cardiovascular disease; NAFLD, non-alcoholic fatty liver disease; NASH, non-alcoholic steatohepatitis; T2DM, type 2 diabetes mellitus.

A bolded value indicates that the lower limit of the odds ratio is greater than 1 and implies that the prevalence of the somatic disease is significantly higher in the ADHD patient group than the control one.

**Figure 3:**
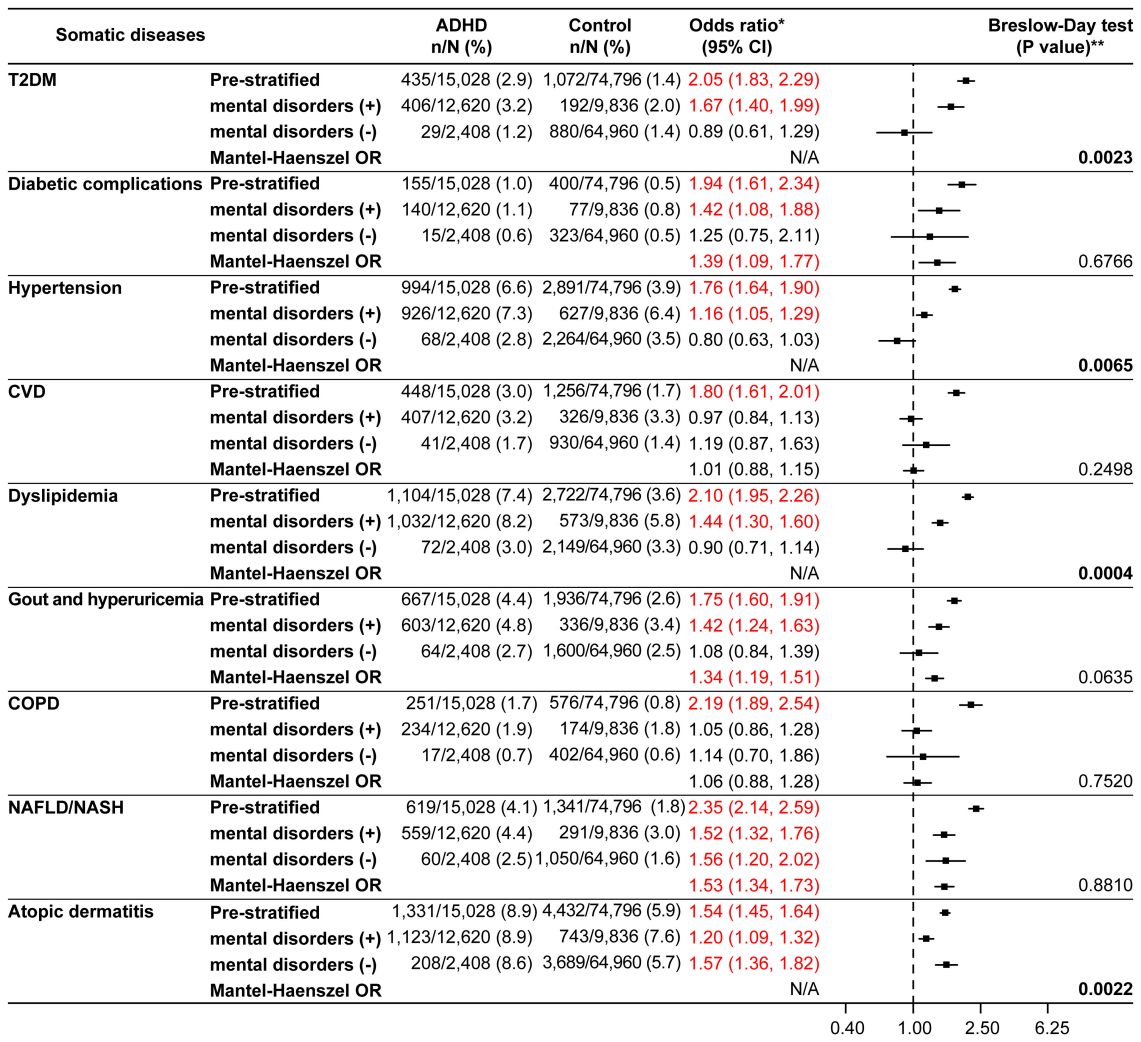
Somatic diseases stratified by the presence or absence of mental disorders (matched analysis set). ^*^Odds ratio for the ADHD/control group in the matched analysis set, including stratification results. ^**^P values in bold font were significant and rejected by the Breslow-Day test. The Mantel-Haenszel odds ratio (Mantel-Haenszel OR) was not indicated if rejected by the Breslow-Day test. ADHD, attention deficit hyperactivity disorder; CI, confidence interval; COPD, chronic obstructive pulmonary disease; CVD, cardiovascular disease; N/A, not applicable; NAFLD, non-alcoholic fatty liver disease; NASH, non-alcoholic steatohepatitis; T2DM, type 2 diabetes mellitus.

Upon further stratification by the presence or absence of mental disorders, the ORs for all somatic diseases of interest except for CVD and COPD were significant in the presence of mental disorders (**Figure 3**). The significant *p* values according to the Breslow-Day test for T2DM (*p*=0.0023), hypertension (*p*=0.0065), dyslipidemia (*p*=0.0004), and atopic dermatitis (*p*=0.0022) indicated a lack of homogeneity between the strata. The stratum-specific ORs for these somatic diseases were different (**Figure 3**). Among the somatic diseases rejected by the Breslow-Day test, the stratum-specific ORs for T2DM, hypertension, and dyslipidemia were in opposite directions across OR=1 (mental disorders: present, OR>1; absent, OR<1), whereas those for atopic dermatitis were in the same direction (OR>1 for both; **Figure 3**). Analysis of the age and sex distributions of the ADHD and control groups stratified by the presence or absence of mental disorders showed differences between the groups in the stratum without mental disorders, where the ADHD group was biased toward younger age (mean age; 28.1 years in the ADHD group versus 32.9 years in the control group, 18–29 years; 66.2% in the ADHD group versus 46.5% in the control group) and males (73.7% in the ADHD group versus 62.1% in the control group) (**Supplementary Table 4**). Logistic regression analyses adjusted for the presence or absence of mental disorders, the presence or absence of mental disorders-age, and the presence or absence of mental disorders-sex (the last two were interaction terms) confirmed significant ORs in the ADHD group for all somatic diseases of interest except CVD and COPD (**Supplementary Figure 1**).

### 3.3 Somatic diseases by age

Older age categories were associated with a higher prevalence of all comorbidities of interest, with the exception of atopic dermatitis (**Table 3**). For all somatic diseases except atopic dermatitis, the prevalence was <2.0% among participants aged 18–29 years (**Table 3**). The prevalence of diabetic complications and COPD was low (<2.9%) compared with that of other comorbidities across all age categories in the study population (**Table 3**).

**Table 3:** Somatic diseases (prevalence and odds ratio) by age categories (matched analysis set).

	18–29 years old			30–39 years old			≥40 years old		
Somatic diseases n (%)	ADHD N=6,912	Control N=34,216	Odds ratio^*^ (95% CI)	ADHD N=3,690	Control N=18,450	Odds ratio^*^ (95% CI)	ADHD N=4,426	Control N=22,130	Odds ratio^*^ (95% CI)
**T2DM**	37 (0.5)	70 (0.2)	**2.63 (1.76, 3.91)**	80 (2.2)	129 (0.7)	**3.15 (2.38, 4.17)**	318 (7.2)	873 (3.9)	**1.88 (1.65, 2.15)**
**Diabetes complications**	13 (0.2)	31 (0.1)	**2.08 (1.09, 3.97)**	23 (0.6)	43 (0.2)	**2.68 (1.62, 4.46)**	119 (2.7)	326 (1.5)	**1.85 (1.49, 2.29)**
**Hypertension**	94 (1.4)	112 (0.3)	**4.20 (3.19, 5.53)**	139 (3.8)	279 (1.5)	**2.55 (2.07, 3.13)**	761 (17.2)	2,500 (11.3)	**1.63 (1.49, 1.78)**
**CVD**	81 (1.2)	233 (0.7)	**1.73 (1.34, 2.23)**	63 (1.7)	166 (0.9)	**1.91 (1.43, 2.56)**	304 (6.9)	857 (3.9)	**1.83 (1.60, 2.10)**
**Dyslipidemia**	83 (1.2)	150 (0.4)	**2.76 (2.11, 3.61)**	201 (5.5)	334 (1.8)	**3.12 (2.61, 3.74)**	820 (18.5)	2,238 (10.1)	**2.02 (1.85, 2.21)**
**Gout and hyperuricemia**	123 (1.8)	231 (0.7)	**2.67 (2.14, 3.32)**	154 (4.2)	392 (2.1)	**2.01 (1.66, 2.43)**	390 (8.8)	1,313 (5.9)	**1.53 (1.36, 1.72)**
**COPD**	69 (1.0)	170 (0.5)	**2.02 (1.52, 2.67)**	60 (1.6)	140 (0.8)	**2.16 (1.59, 2.93)**	122 (2.8)	266 (1.2)	**2.33 (1.88, 2.89)**
**NAFLD/NASH**	134 (1.9)	168 (0.5)	**4.01 (3.19, 5.04)**	165 (4.5)	357 (1.9)	**2.37 (1.97, 2.86)**	320 (7.2)	816 (3.7)	**2.04 (1.78, 2.33)**
**Atopic dermatitis**	731 (10.6)	2,609 (7.6)	**1.43 (1.31, 1.56)**	327 (8.9)	915 (5.0)	**1.86 (1.63, 2.13)**	273 (6.2)	908 (4.1)	**1.54 (1.34, 1.77)**

^*^ Odds ratio for the ADHD/control group in the matched analysis set.

ADHD, attention deficit hyperactivity disorder; CI, confidence interval; COPD, chronic obstructive pulmonary disease; CVD, cardiovascular disease; NAFLD, non-alcoholic fatty liver disease; NASH, non-alcoholic steatohepatitis; T2DM, type 2 diabetes mellitus.

A bolded value indicates that the lower limit of the odds ratio is greater than 1 and implies that the prevalence of the somatic disease is significantly higher in the ADHD patient group than the control one.

**Figure 4:**
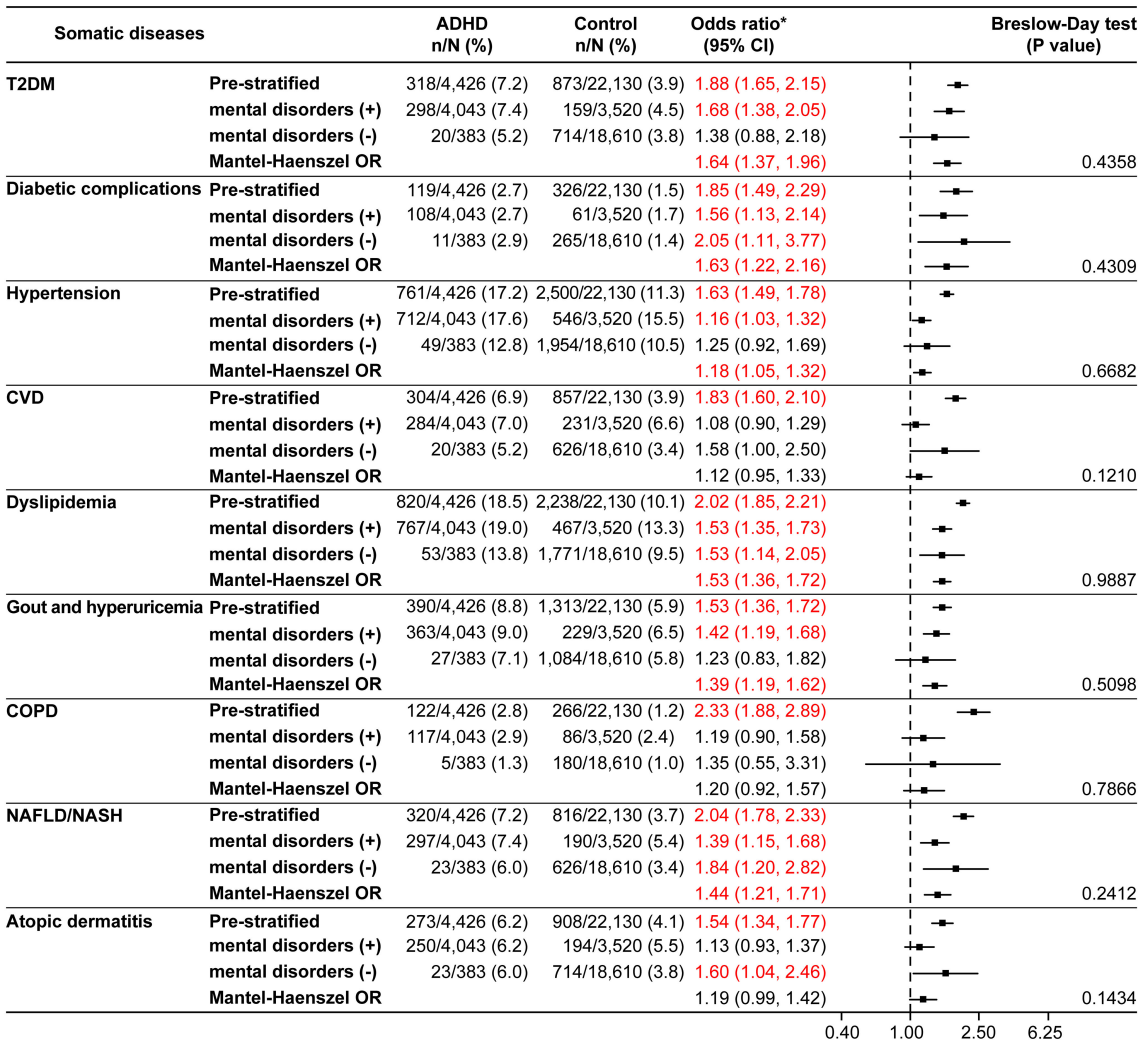
Somatic diseases stratified by the presence or absence of mental disorders in the ≥40 years age category (matched analysis set). ^*^Odds ratio for the ADHD/control group in the matched analysis set, including stratification results. ADHD, attention deficit hyperactivity disorder; CI, confidence interval; COPD, chronic obstructive pulmonary disease; CVD, cardiovascular disease; NAFLD, non-alcoholic fatty liver disease; NASH, non-alcoholic steatohepatitis; OR, odds ratio; T2DM, type 2 diabetes mellitus.

The null hypotheses of CVD (*p*=0.0179) and NAFLD/NASH (*p*=0.0221) were rejected by the Breslow-Day test in the 18–29 years age category (**Supplementary Figure 2**), whereas there were no rejections by the Breslow-Day test in the other age categories (**Supplementary Figure 3**, **Figure 4**). For T2DM, hypertension, dyslipidemia, and gout and hyperuricemia, the Mantel-Haenszel ORs were significantly higher in all age categories. In the ≥40 years age category, the Mantel-Haenszel ORs were significant for all somatic diseases except CVD, COPD, and atopic dermatitis (**Figure 4**).

### 3.4 Somatic diseases by sex

T2DM, diabetic complications, hypertension, dyslipidemia, gout and hyperuricemia, and NAFLD/NASH were more common in men than in women in both the ADHD and control groups (**Table 4**). The ORs for the ADHD versus control group for all somatic diseases except atopic dermatitis and COPD were higher in women than in men.

**Table 4:** Somatic diseases (prevalence and odds ratio) by sex (matched analysis set).

	Female			Male		
Somatic diseases n (%)	ADHD N=5,903	Control N=29,171	Odds ratio^*^ (95% CI)	ADHD N=9,125	Control N=44,285	Odds ratio^*^ (95% CI)
**T2DM**	146 (2.5)	295 (1.0)	**2.48 (2.03, 3.03)**	289 (3.2)	777 (1.7)	**1.89 (1.65, 2.16)**
**Diabetes complications**	53 (0.9)	124 (0.4)	**2.12 (1.54, 2.93)**	102 (1.1)	276 (0.6)	**1.86 (1.48, 2.33)**
**Hypertension**	305 (5.2)	848 (2.9)	**1.82 (1.59, 2.08)**	689 (7.6)	2,043 (4.5)	**1.74 (1.59, 1.91)**
**CVD**	187 (3.2)	477 (1.6)	**1.97 (1.66, 2.34)**	261 (2.9)	779 (1.7)	**1.70 (1.47, 1.95)**
**Dyslipidemia**	364 (6.2)	778 (2.7)	**2.40 (2.11, 2.73)**	740 (8.1)	1,944 (4.3)	**1.98 (1.82, 2.17)**
**Gout and hyperuricemia**	73 (1.2)	145 (0.5)	**2.51 (1.89, 3.33)**	594 (6.5)	1,791 (3.9)	**1.70 (1.55, 1.88)**
**COPD**	120 (2.0)	284 (1.0)	**2.11 (1.70, 2.62)**	131 (1.4)	292 (0.6)	**2.26 (1.84, 2.78)**
**NAFLD/NASH**	165 (2.8)	305 (1.1)	**2.72 (2.25, 3.30)**	454 (5.0)	1,036 (2.3)	**2.25 (2.01, 2.52)**
**Atopic dermatitis**	533 (9.0)	1,867 (6.4)	**1.45 (1.31, 1.61)**	798 (8.8)	2,565 (5.6)	**1.61 (1.48, 1.75)**

^*^ Odds ratio for the ADHD/control group in the matched analysis set.

ADHD, attention deficit hyperactivity disorder; CI, confidence interval; COPD, chronic obstructive pulmonary disease; CVD, cardiovascular disease; NAFLD, non-alcoholic fatty liver disease; NASH, non-alcoholic steatohepatitis; T2DM, type 2 diabetes mellitus.

A bolded value indicates that the lower limit of the odds ratio is greater than 1 and implies that the prevalence of the somatic disease is significantly higher in the ADHD patient group than the control one.

### 3.5 Sensitivity analyses

In the ADHD group, there were 13,829 patients having yearly medical records for ADHD, of whom 11,505 patients had both medical records for ADHD and dispensing dates for ADHD medication, and matched control sets were 68,951 and 57,487 participants, respectively. There were no differences in characteristics between the main and sensitivity analyses (**Supplementary Table 5**). For somatic diseases, the main and sensitivity analyses yielded almost the same results regardless of stratification, whereas the null hypothesis for hypertension was not rejected but for CVD was rejected by the Breslow-Day test in one sensitivity analysis with the ADHD group having yearly medical records for both ADHD and dispensing dates for ADHD medication. (**Supplementary Table 6**, **Supplementary Figures 4, 5**).

## 4 Discussion

In our study, we observed a high prevalence of comorbid systemic disorders, including chronic diseases, in adult patients with ADHD, indicating that not only mental disorders but also somatic diseases could be prevalent in adult patients with ADHD. Thus, our results demonstrate that ADHD may be associated with disease burden arising from both ADHD and non-ADHD comorbidities. The outcomes from our study have the potential to enhance societal awareness toward ADHD and its associated burden, contributing to improved understanding of the challenges and impact of ADHD. Despite progress in the understanding of ADHD in Japan, apprehensions about negative social associations with ADHD still persist (23) and diagnosed adults are known to experience self-stigma associated with their diagnosis (24).

The prevalence of most somatic diseases increased with advancing age, and association of several somatic diseases with ADHD became more pronounced in the presence of mental disorders. In patients aged ≥40 years, the results of stratified analysis showed high ORs for all somatic diseases except CVD, COPD, and atopic dermatitis. In addition, ADHD was associated with chronic systemic diseases, particularly lifestyle disorders such as T2DM, hypertension, and dyslipidemia, whose prevalence increases with age, highlighting the importance of lifestyle management and careful monitoring of comorbidities in patients with ADHD as their age advances. The results from our study might provide a deeper understanding on the possibility of the complex interplay between mental disorders and somatic diseases in patients with ADHD. The additional insights emphasize a need for greater collaboration between psychiatrists and other specialists to prevent the onset of somatic diseases and timely identify and manage these conditions in patients with ADHD and other mental disorders.

In the ADHD group, according to the distribution of the initial year of ADHD diagnosis, the number of patients increased in the later years of the data period, which is understandable, given the 21.1-fold increase in the incidence of ADHD among Japanese adults over the past decade (6). This finding could be attributed to the fact that it has become increasingly easier to diagnose patients with ADHD instead of an increase in patients with true ADHD. The incidence of ADHD reportedly plateaued to approximately 6/10,000 person-years in 2018 and became comparable with the incidence of ADHD among Asian adults residing in the US in 2016 (6.88/10,000 person-years) (6), which may imply that the sensitivity of ADHD diagnosis among Japanese adults has reached a standard level (6). During the same period in Japan, DSM-5 was introduced and atomoxetine (in August 2012), methylphenidate (in December 2013), and guanfacine (in June 2019) were approved for the treatment of ADHD in adults, which may have also increased disease awareness. In addition, it could be mentioned only for the ADHD patient population in this study that the number of enrollees in the JMDC database has slightly increased over the years (except for 2014, when the number of health insurances JMDC Inc. contracted was small).

The ADHD population was younger (mean age: 33.1 years) than the pre-matched control population (mean age: 45.5 years), which may suggest that it could be challenging for patients with ADHD to remain employed. The number of men in both groups was high (>60.0%), which could be due to the characteristics of the JMDC database, a health insurance claims database that covers employees and, therefore, may comprise a greater proportion of men than women. This is consistent with the results of an epidemiological survey for ADHD in Japan that reported no notable differences in the estimated prevalence of adult ADHD between men and women (25). However, a preponderance of ADHD in men has been reported in a systematic review of studies investigating the gender-based prevalence of ADHD (26).

The odds for occurrence of all comorbidities of interest, including lifestyle disorders, were significantly higher in participants with ADHD than in those without ADHD in our study. The prevalence of T2DM and hypertension observed among adults with ADHD in our study was 2.9% and 6.6%, respectively, which is comparable with that observed in a previous large Swedish national register study conducted in patients with ADHD (T2DM, 3.9%; hypertension, 8.5%) (15). These slight discrepancies in prevalence might be partially due to differences in the duration of the assessment period. Consistent with our results, both T2DM and hypertension were more common in men than in women with ADHD in the Swedish study. The prevalence of T2DM in patients with ADHD in a pooled analysis of five studies was higher than that in the control population (OR: 2.05, 95% CI: 1.37, 3.07, *I* ^2^ = 92%, *p*<0.01) (16). Moreover, a Taiwanese study showed a higher incidence of T2DM in young adults with ADHD (18). On stratified analysis in our study, the Breslow-Day test rejected the homogeneity of the three components of metabolic syndrome, and the stratum-specific OR was >1 in the presence of mental disorders but <1 in the absence of mental disorders, suggesting that mental disorders may enhance the association between ADHD and T2DM, hypertension, and dyslipidemia. Previous research suggests that ADHD itself is not related to chronic disorders, unlike depressive symptoms or affective disorders (19, 27–30). However, stratum-specific OR of <1 and >1 in the absence and presence of mental disorders, respectively, for chronic disorders in our study, imply that ADHD could potentially be an interacting factor that enhances the association between certain mental disorders and components of metabolic syndrome. Alternatively, this may be due to the fact that patients with ADHD with mental disorders were relatively young. Subgroup analyses by age category showed significantly higher Mantel-Haenszel ORs for all age categories for these disorders. For CVD and COPD, although the ORs were significantly higher in the ADHD group on non-stratified analysis in the present study, no significant results were derived from stratified analysis and logistic regression analysis, suggesting that mental disorders may be mediators for CVD and COPD. Previous studies regarding CVD have shown inconsistent results (11, 31). On the other hand, there is a previous registry study that reported a 3.2 times higher odds of having COPD in patients with ADHD than in those without ADHD (10). Furthermore, previous reports have indicated that genetic susceptibility to ADHD could have a partial causative role in COPD development (32) and that young patients with ADHD have a greater tendency to start smoking (33), which is a risk factor for COPD. It might be necessary to consider that the prevalence (1.7%) was low for COPD. Therefore, the association between ADHD and CVD and COPD warrants further investigation.

The Mantel-Haenszel OR was >1 and significant for diabetic complications (1.39; 95% CI: 1.09, 1.77); however, the prevalence (1.0%) was low in our study. Nevertheless, the significant ORs for diabetic complications suggest that ADHD characteristics could negatively impact treatment adherence in patients with comorbid T2DM, leading to T2DM progression and additional complications.

The OR for NAFLD/NASH (2.35; 95% CI: 2.14, 2.59) in patients with ADHD versus the control group was the highest among all somatic diseases reported in our study. Lifestyle factors may play an underlying role in the development of NAFLD/NASH (34), similar to that observed in T2DM, hypertension, and dyslipidemia. However, in contrast to T2DM, hypertension, and dyslipidemia, the Mantel-Haenszel OR (1.53; 95% CI: 1.34, 1.73) in our study demonstrated an association between NAFLD/NASH and ADHD regardless of mental disorders. This difference could have arisen from the fact that NAFLD is a continuum, with obesity as a precursor of NAFLD, whereas metabolic syndrome and T2DM develop gradually following NAFLD (34). Intestinal microbiota, a suspected common pathological factor for both ADHD and NAFLD/NASH (34), could be another reason for the significant association observed between ADHD and NAFLD/NASH in our analysis. The Mantel-Haenszel OR for gout and hyperuricemia (1.34; 95% CI: 1.19, 1.51) was consistently and significantly higher than 1 through all subgroup analyses and sensitivity analyses, which also suggested an association with ADHD. A previous genome-wide association study has reported a significant genetic correlation between gout and hyperuricemia, and ADHD (14).

The findings of the Breslow-Day test in our study show that atopic dermatitis may have interacted with mental disorders. On evaluation of the stratum-specific ORs, those for atopic dermatitis were significant in both patients with and without mental disorders, suggesting an association between this comorbidity and ADHD, irrespective of the presence or absence of mental disorders. This view is also likely supported by the results of the logistic regression analysis. For atopic dermatitis, the OR was higher among those without mental disorders (1.57; 95% CI: 1.36, 1.82) than among those with mental disorders (1.20; 95% CI: 1.09, 1.32) and could be attributed to the age difference; the obvious difference between the ORs may have led to rejection by the Breslow-Day test. The symptoms of atopic dermatitis and increased sensitivity to stimuli arising from the upregulation of neuroimmune factors, leading to sleep disturbances, have been previously hypothesized to increase the risk of ADHD in patients with atopic dermatitis (12). Neuroimmunological pathways and psychological stress from atopic diseases have been reported to increase vulnerability to ADHD (35). According to such reports, atopic diseases may impact the severity of ADHD symptoms. Moreover, a previous study showed significantly higher odds (OR=1.48) of the occurrence of ADHD in patients with atopic dermatitis than in those without, alluding to an association between these conditions (13).

ORs were higher in women than in men for all comorbidities except COPD and atopic dermatitis. It is possible that the severity of ADHD was greater in women than in men in our study, given that women were older and had a higher prevalence of mental disorders than men. However, previous evidence indicates that diagnosis and treatment are possibly delayed in women with ADHD and other mental disorders likely coexist (36).

## 5 Strengths and limitations

Our study has several strengths. The importance of demonstrating the association between ADHD and comorbidities may increase with age, as is evident from our results, which demonstrated the highest prevalence of chronic diseases in both the ADHD and control groups among participants aged ≥40 years. As the JMDC database is primarily for individuals with health insurance and covers employees of large companies and their dependents, its use allowed us to focus on the patient population aged ≥40 years. Further, as long as an individual does not withdraw from health insurances, we can obtain all receipt information including hospitalization, Diagnosis Procedure Combination, outpatient, and dispensing for him/her. In addition, we were able to collect data on several comorbidities, potentially because the universal health insurance system in Japan allows patients to consult their doctors even in the case of mild symptoms and to reach an early diagnosis. Furthermore, stratified analysis (versus multivariate analysis) in the presence or absence of mental disorders conducted to investigate their association has several advantages, including that it makes both the investigator and readers aware of the data distribution by key study variables, and reduces the likelihood of the occurrence of some bias because analysis models are not required (37). Therefore, according to Rothman, the first step should always be a stratified analysis, and multivariate analysis ought to be thought of as a supplement to it (37). Following this policy, we performed a supplementary logistic regression analysis in addition to the stratified analysis. Stratified analyses based on the presence or absence of mental disorders were also important, given that most participants (84%) with ADHD reported mental disorders compared with approximately 19% of those without ADHD. Moreover, the ADHD group may have had different mental disorders than the control population. Although we had the option of including individuals without mental disorders, this approach could have led to the selection of a unique patient group in the ADHD population.

ADHD is a developmental disorder (38), and disease characteristics may be present at birth. In Japan, screening for developmental disorders, including childhood ADHD, is optional despite the availability of screening tools for ADHD in children, which may often lead to a missed diagnosis of ADHD in children (39). The developmental nature of ADHD makes it challenging to investigate the longitudinal relationship of ADHD diagnosis and onset of comorbidities as setting an index date for the ADHD group (i.e., the date of the first diagnosis of ADHD) in a cohort study design could raise the concern of missing the true outcome information during the look-back period. Thus, a cross-sectional study design was adopted. The cross-sectional study design can also limit the investigation of causality between ADHD and somatic diseases of interest, as the time of ADHD diagnosis, drug dispensing, or onset of mental disorders or somatic diseases of interest could not be ascertained. Among the mental disorders, there are cases in which the medication drugs may be risk factors for somatic diseases. However, due to the above reason, the effects of treatment could not be investigated in this study. Thus, the outcomes should be interpreted cautiously, and causal inferences and definitive claims should be avoided. Additionally, this was an exploratory study with the aim of assessing the prevalence of somatic diseases in patients with ADHD and comparing it with that in a control population, stratified by mental disorders. Therefore, further studies are warranted to investigate the causal relationship between ADHD and somatic diseases. Another limitation in the methodology was that the observation period for the control population had to be started earlier compared with that for the ADHD population to allow for the investigation of their data over a longer period to rule out any history of ADHD diagnosis or medication. An observation period starting at the same time for the ADHD population could have increased the likelihood of excluding several cases with ADHD and including relatively healthy cases or cases with milder ADHD due to the persistent enrollment of such cases in the insurance system, leading to biased outcomes. Furthermore, the ICD-10 code definitions may not be entirely precise in identifying mental disorders and somatic diseases, which could impact the outcomes; some definitions for mental disorders may include acute stress reactions.

Our results could have underestimated the association between ADHD and somatic diseases, given that the JMDC database includes only employees with health insurance and their dependents, and may exclude dependent persons, particularly those who were previously employed but who were unemployed during the data period. In addition, the study population may have primarily comprised patients with relatively mild ADHD, as it could be challenging for patients with severe disease to seek frequent medical consultation. Thus, more pronounced results could be obtained if data are collected in routine clinical practice. Moreover, the higher odds of occurrence of several comorbidities in the ADHD group versus the control group could be attributed to greater awareness among both patients and treating physicians, and to more frequent health checkups due to ADHD leading to the diagnosis of these conditions. However, the significantly higher ORs for a majority of somatic diseases on stratified analysis suggest that there was possibly no difference in the frequency of hospital visits between the ADHD and the control group for the stratum with mental disorders. Additionally, the impact of unmeasured confounding factors in the database on the study results cannot be ruled out; therefore, the results should be interpreted with caution.

## 6 Conclusion

The analysis of a health insurance claims database in Japan suggested that the prevalence of several somatic diseases is higher in adults with ADHD than in those without ADHD, which may lead to additional disease burden in patients with ADHD. Patients with ADHD need to be closely monitored, especially those with mental disorders. Furthermore, the prevalence of chronic diseases was high in relatively older (aged ≥40 years) individuals with ADHD; therefore, future research should focus on this patient population.

## Data availability statement

The original contributions presented in the study are included in the article/**Supplementary Material**. Further inquiries can be directed to the corresponding author.

## Ethics statement

This study was conducted in compliance with the Ethical Guidelines for Medical and Health Research Involving Human Subjects and its Guidance document (20). The studies were conducted in accordance with the local legislation and institutional requirements. Informed consent was not required because the JMDC database contains Anonymized Processed Information, as defined in Article 2, Paragraph 6 of the Act on the Protection of Personal Information (Act No. 57 of 2003) (21). This study (UMIN000049401) is registered at the UMIN Clinical Trials Registry (https://www.umin.ac.jp/ctr/).

## Author contributions

YT, YS, SI, PT, HM, YI, and TK conceived and designed the study. SI and HM performed the analyses. YT, YS, SI, PT, HM, YI, and TK interpreted the data. SI and HM accessed and verified the data. YT, YS, SI, and YI were involved in the writing of the manuscript. YT, YS, SI, PT, HM, YI, and TK were responsible for the decision to submit the manuscript for publication. All authors contributed to the article and approved the submitted version.
