# Supplementary material for: Prevalence of somatic diseases in adults with attention deficit hyperactivity disorder in Japan is highest in people aged ≥40 years with mental disorders: a cross-sectional study of a Japanese health insurance claims database
Source: Front Psychiatry. 2024 Feb 14;15:1197513. doi: 10.3389/fpsyt.2024.1197513 (PMC10899330; doi:10.3389/fpsyt.2024.1197513)
Supplement: Supplementary file 1 [file DataSheet_1.docx]

Supplementary Material

**Prevalence of Somatic Diseases in Adults With Attention Deficit Hyperactivity Disorder in Japan** **is Highest in People Aged ≥40 Years With Mental Disorders: A Cross-sectional Study of a Japanese Health Insurance Claims Database**

Yoshikazu Takaesu^1^, Yumi Sato^2^, Shinpei Iwata^3^, Patcharapim Takizawa^4^, Hideyuki Miyauchi^3^, Yoshikazu Ishimoto^2^* and Tsuyoshi Kondo^1^

^1^Department of Neuropsychiatry, Graduate School of Medicine, University of the Ryukyus, Okinawa, Japan.

^2^Medical Affairs Department, Integrated Disease Care Division, Shionogi & Co., Ltd., Osaka, Japan.

^3^PMS & Pharmacoepidemiology Department, Shionogi Pharmacovigilance Center Co., Ltd., Osaka, Japan.

^4^Japan Medical Office, Takeda Pharmaceutical Co., Ltd., Tokyo, Japan.

***Correspondence:**Yoshikazu Ishimoto
[yoshikazu.ishimoto@shionogi.co.jp](mailto:yoshikazu.ishimoto@shionogi.co.jp)

# Supplementary tables

**Supplementary Table 1.** Outcome definitions.

| **Outcome** | **Definition** |
| --- | --- |
| T2DM | A medical record for T2DM (ICD-10 codes: E11, E14; no suspicion flag) in a month during the assessment period and dispensing date for T2DM medication (ATC codes: A10C, A10H, A10J, A10K, A10L, A10M, A10N, A10P, A10S, A10X) within the same month (1, 2). |
| Diabetes complications | In addition to the definition of T2DM, the following definitions were used to identify diabetic complications among participants. Medical records for diabetic nephropathy (ICD-10 codes: E102, E112, E142; no suspicion flag), diabetic retinopathy (ICD-10 codes: E103, E113, E143; no suspicion flag), or diabetic neuropathy (ICD-10 codes: E104, E114; no suspicion flag) in a month during the assessment period (3, 4). |
| Hypertension | A medical record for hypertension (ICD-10 codes: I10-I15; no suspicion flag) in a month during the assessment period and dispensing date for hypertension medication (ATC codes: C02, C03, C04, C07, C08, and C09) within the same month (5). |
| CVD | A medical record for myocardial infarction (ICD-10 code: I210, I211, I212, I213, I214, I219; no suspicion flag), angina pectoris (ICD-10 code: I200, I201, I208, I209; no suspicion flag), stroke (ICD-10 codes: I600, I601, I602, I603, I604, I605, I606, I607, I608, I609, I610, I611, I613, I614, I615, I616, I619, I629, I630, I631, I632, I633, I634, I635, I636, I638, I639; no suspicion flag), heart failure (ICD-10 codes: I500, I501, I509, I110; no suspicion flag), or atrial fibrillation (ICD-10 code: I480, I481, I482, I483, I484, I489; no suspicion flag) during the assessment period (6). |
| Dyslipidemia | A medical record for dyslipidemia (ICD-10 code: E78; no suspicion flag) in a month during the assessment period and a dispensing date for dyslipidemia medication (ATC code: C10) within the same month (7). |
| Gout and hyperuricemia | A medical record for gout (ICD-10 code: M10; no suspicion flag) or hyperuricemia (ICD-10 code: E790; no suspicion flag) in a month during the assessment period (8). |
| COPD | A medical record for chronic bronchitis (ICD-10 code: J42; no suspicion flag), emphysema (ICD-10 code: J43; no suspicion flag), or COPD (ICD-10 code: J44; no suspicion flag) in a month during the assessment period (9). |
| NAFLD/NASH | A medical record for NAFLD (ICD-10 code: K760; no suspicion flag) or NASH (ICD-10 code: K758; no suspicion flag) in a month during the assessment period (10). |
| Atopic dermatitis | A medical record for atopic dermatitis (ICD-10 code: L20; no suspicion flag) in a month during the assessment period (11). |

ATC, Anatomical Therapeutic Chemical; COPD, chronic obstructive pulmonary disease; CVD, cardiovascular disease; ICD-10, International Classification of Diseases, Tenth Edition; NAFLD, non-alcoholic fatty liver disease; NASH, non-alcoholic steatohepatitis; T2DM, type 2 diabetes mellitus.

**Supplementary Table 2.** Population demographics and characteristics by age category (matched analysis set).

|  | **18–29 years** | | **30–39 years** | | **≥40 years** | |
| --- | --- | --- | --- | --- | --- | --- |
| **Variables** | **ADHD**  **N=6,912**  **n (%)** | **Control**  **N=34,216**  **n (%)** | **ADHD**  **N=3,690**  **n (%)** | **Control**  **N=18,450**  **n (%)** | **ADHD**  **N=4,426**  **n (%)** | **Control**  **N=22,130**  **n (%)** |
| **Age, years** | | | | | | |
| **Mean ± SD** | 23.1 ± 3.5 | 23.0 ± 3.5 | 34.1 ± 2.9 | 34.1 ± 2.9 | 48.0 ± 5.8 | 48.0 ± 5.8 |
| **Sex** | | | | | | |
| **Female** | 2,569 (37.2) | 12,501 (36.5) | 1,426 (38.6) | 7,130 (38.6) | 1,908 (43.1) | 9,540 (43.1) |
| **Male** | 4,343 (62.8) | 21,715 (63.5) | 2,264 (61.4) | 11,320 (61.4) | 2,518 (56.9) | 12,590 (56.9) |
| **Initial year of ADHD diagnosis** | | | | | | |
| **2013 (June–December)** | 337 (4.9) | - | 36 (1.0) | - | 62 (1.4) | - |
| **2014** | 99 (1.4) | - | 40 (1.1) | - | 64 (1.4) | - |
| **2015** | 327 (4.7) | - | 128 (3.5) | - | 216 (4.9) | - |
| **2016** | 408 (5.9) | - | 233 (6.3) | - | 398 (9.0) | - |
| **2017** | 642 (9.3) | - | 435 (11.8) | - | 597 (13.5) | - |
| **2018** | 851 (12.3) | - | 624 (16.9) | - | 740 (16.7) | - |
| **2019** | 1,275 (18.4) | - | 751 (20.4) | - | 873 (19.7) | - |
| **2020** | 1,888 (27.3) | - | 964 (26.1) | - | 963 (21.8) | - |
| **2021 (January–September)** | 1,085 (15.7) | - | 479 (13.0) | - | 513 (11.6) | - |
| **Start year of observation period** | | | | | | |
| **<2013** | 688 (10.0) | 19,817 (57.9) | 273 (7.4) | 10,470 (56.7) | 638 (14.4) | 13,721 (62.0) |
| **2013** | 488 (7.1) | 14,399 (42.1) | 244 (6.6) | 7,980 (43.3) | 404 (9.1) | 8,409 (38.0) |
| **2014** | 110 (1.6) | 0 (0) | 97 (2.6) | 0 (0) | 71 (1.6) | 0 (0) |
| **2015** | 733 (10.6) | 0 (0) | 468 (12.7) | 0 (0) | 716 (16.2) | 0 (0) |
| **2016** | 763 (11.0) | 0 (0) | 471 (12.8) | 0 (0) | 607 (13.7) | 0 (0) |
| **2017** | 939 (13.6) | 0 (0) | 561 (15.2) | 0 (0) | 596 (13.5) | 0 (0) |
| **2018** | 1,016 (14.7) | 0 (0) | 565 (15.3) | 0 (0) | 529 (12.0) | 0 (0) |
| **2019** | 1,105 (16.0) | 0 (0) | 575 (15.6) | 0 (0) | 468 (10.6) | 0 (0) |
| **2020** | 1,070 (15.5) | 0 (0) | 436 (11.8) | 0 (0) | 397 (9.0) | 0 (0) |
| **Number of mental disorders** | | | | | | |
| **0** | 1,594 (23.1) | 30,194 (88.2) | 431 (11.7) | 16,156 (87.6) | 383 (8.7) | 18,610 (84.1) |
| **≥1** | 5,318 (76.9) | 4,022 (11.8) | 3,259 (88.3) | 2,294 (12.4) | 4,043 (91.3) | 3,520 (15.9) |

ADHD, attention deficit hyperactivity disorder; SD, standard deviation.

**Supplementary Table 3.** Population demographics and characteristics by sex (matched analysis set).

|  | **Female** | | **Male** | |
| --- | --- | --- | --- | --- |
| **Variables** | **ADHD**  **N=5,903**  **n (%)** | **Control**  **N=29,171**  **n (%)** | **ADHD**  **N=9,125**  **n (%)** | **Control**  **N=45,625**  **n (%)** |
| **Age, years** | | | | |
| **Mean ± SD** | 33.9 ± 11.4 | 34.0 ± 11.4 | 32.7 ± 11.4 | 32.7 ± 11.4 |
| **18–29 years** | 2,569 (43.5) | 12,501 (42.9) | 4,343 (47.6) | 21,715 (47.6) |
| **30–39 years** | 1,426 (24.2) | 7,130 (24.4) | 2,264 (24.8) | 11,320 (24.8) |
| **≥40 years** | 1,908 (32.3) | 9,540 (32.7) | 2,518 (27.6) | 12,590 (27.6) |
| **Initial year of ADHD diagnosis** | | | | |
| **2013 (June–December)** | 103 (1.7) | - | 332 (3.6) | - |
| **2014** | 55 (0.9) | - | 148 (1.6) | - |
| **2015** | 210 (3.6) | - | 461 (5.1) | - |
| **2016** | 332 (5.6) | - | 707 (7.7) | - |
| **2017** | 567 (9.6) | - | 1,107 (12.1) | - |
| **2018** | 848 (14.4) | - | 1,367 (15.0) | - |
| **2019** | 1,200 (20.3) | - | 1,699 (18.6) | - |
| **2020** | 1,650 (28.0) | - | 2,165 (23.7) | - |
| **2021 (January–September)** | 938 (15.9) | - | 1,139 (12.5) | - |
| **Start year of observation period** | | | | |
| **<2013** | 549 (9.3) | 17,087 (58.6) | 1,050 (11.5) | 26,921 (59.0) |
| **2013** | 388 (6.6) | 12,084 (41.4) | 748 (8.2) | 18,704 (41.0) |
| **2014** | 111 (1.9) | 0 (0) | 167 (1.8) | 0 (0) |
| **2015** | 725 (12.3) | 0 (0) | 1,192 (13.1) | 0 (0) |
| **2016** | 717 (12.1) | 0 (0) | 1,124 (12.3) | 0 (0) |
| **2017** | 808 (13.7) | 0 (0) | 1,288 (14.1) | 0 (0) |
| **2018** | 839 (14.2) | 0 (0) | 1,271 (13.9) | 0 (0) |
| **2019** | 898 (15.2) | 0 (0) | 1,250 (13.7) | 0 (0) |
| **2020** | 868 (14.7) | 0 (0) | 1,035 (11.3) | 0 (0) |
| **Number of mental disorders** | | | | |
| **0** | 634 (10.7) | 24,594 (84.3) | 1,774 (19.4) | 40,366 (88.5) |
| **≥1** | 5,269 (89.3) | 4,577 (15.7) | 7,351 (80.6) | 5,259 (11.5) |

ADHD, attention deficit hyperactivity disorder; SD, standard deviation.

**Supplementary Table 4.** Age and sex distributions of the ADHD and control groups stratified by the presence or absence of mental disorders.

|  |  | | **≥1 mental disorders** | | **No mental disorders** | |
| --- | --- | --- | --- | --- | --- | --- |
| **Variables** | **ADHD**  **N=15,028**  **n (%)** | **Control**  **N=74,796**  **n (%)** | **ADHD**  **N=12,620**  **n (%)** | **Control**  **N=9,836**  **n (%)** | **ADHD**  **N=2,408**  **n (%)** | **Control**  **N=64,960**  **n (%)** |
| **Age, years** | | | | |  |  |
| **Mean ± SD** | 33.1 ± 11.4 | 33.2 ± 11.4 | 34.1 ± 11.4 | 35.0 ± 11.6 | 28.1 ± 10.1 | 32.9 ± 11.4 |
| **18–29 years** | 6,912 (46.0) | 34,216 (45.7) | 5,318 (42.1) | 4,022 (40.9) | 1,594 (66.2) | 30,194 (46.5) |
| **30–39 years** | 3,690 (24.6) | 18,450 (24.7) | 3,259 (25.8) | 2,294 (23.3) | 431 (17.9) | 16,156 (24.9) |
| **≥40 years** | 4,426 (29.5) | 22,130 (29.6) | 4,043 (32.0) | 3,520 (35.8) | 383 (15.9) | 18,610 (28.6) |
| **Sex** | | | | |  |  |
| **Female** | 5,903 (39.3) | 29,171 (39.0) | 5,269 (41.8) | 4,577 (46.5) | 634 (26.3) | 24,594 (37.9) |
| **Male** | 9,125 (60.7) | 45,625 (61.0) | 7,351 (58.3) | 5,259 (53.5) | 1,774 (73.7) | 40,366 (62.1) |

ADHD, attention deficit hyperactivity disorder; SD, standard deviation.

**Supplementary Table 5.** Characteristics of the ADHD and control groups in the main and sensitivity analysis.

|  |  | | **Sensitivity analysis_1*** | | **Sensitivity analysis_2**** | |
| --- | --- | --- | --- | --- | --- | --- |
| **Variables** | **ADHD**  **N=15,028**  **n (%)** | **Control**  **N=74,796**  **n (%)** | **ADHD**  **N=13,829**  **n (%)** | **Control**  **N=68,951**  **n (%)** | **ADHD**  **N=11,505**  **n (%)** | **Control**  **N=57,487**  **n (%)** |
| **Age, years** | | | | | | |
| **Mean ± SD** | 33.1 ± 11.4 | 33.2 ± 11.4 | 33.3 ± 11.4 | 33.4 ± 11.4 | 33.3 ± 11.2 | 33.3 ± 11.2 |
| **18–29 years** | 6,912 (46.0) | 34,216 (45.7) | 6,249 (45.2) | 31,051 (45.0) | 5,196 (45.2) | 25,942 (45.1) |
| **30–39 years** | 3,690 (24.6) | 18,450 (24.7) | 3,412 (24.7) | 17,060 (24.7) | 2,916 (25.3) | 14,580 (25.4) |
| **≥40 years** | 4,426 (29.5) | 22,130 (29.6) | 4,168 (30.1) | 20,840 (30.2) | 3,393 (29.5) | 16,965 (29.5) |
| **Sex** | | | | | | |
| **Female** | 5,903 (39.3) | 29,171 (39.0) | 5,442 (39.4) | 27,016 (39.2) | 4,456 (38.7) | 22,242 (38.7) |
| **Male** | 9,125 (60.7) | 45,625 (61.0) | 8,387 (60.6) | 41,935 (60.8) | 7,049 (61.3) | 35,245 (61.3) |
| **Initial year of ADHD diagnosis** | | | | | | |
| **2013 (June–December)** | 435 (2.9) | - | 350 (2.5) | - | 235 (2.0) | - |
| **2014** | 203 (1.4) | - | 167 (1.2) | - | 102 (0.9) | - |
| **2015** | 671 (4.5) | - | 578 (4.2) | - | 420 (3.7) | - |
| **2016** | 1,039 (6.9) | - | 918 (6.6) | - | 680 (5.9) | - |
| **2017** | 1,674 (11.1) | - | 1,503 (10.9) | - | 1,147 (10.0) | - |
| **2018** | 2,215 (14.7) | - | 2,025 (14.6) | - | 1,610 (14.0) | - |
| **2019** | 2,899 (19.3) | - | 2,706 (19.6) | - | 2,180 (18.9) | - |
| **2020** | 3,815 (25.4) | - | 3,505 (25.3) | - | 3,059 (26.6) | - |
| **2021 (January–September)** | 2,077 (13.8) | - | 2,077 (15.0) | - | 2,072 (18.0) | - |
| **Start year of observation period** | | | | | | |
| **<2013** | 1,599 (10.6) | 44,008 (58.8) | 1,417 (10.2) | 40,689 (59.0) | 1,066 (9.3) | 33,772 (58.7) |
| **2013** | 1,136 (7.6) | 30,788 (41.2) | 995 (7.2) | 28,262 (41.0) | 759 (6.6) | 23,715 (41.3) |
| **2014** | 278 (1.8) | 0 (0) | 249 (1.8) | 0 (0) | 190 (1.7) | 0 (0) |
| **2015** | 1,917 (12.8) | 0 (0) | 1,750 (12.7) | 0 (0) | 1,368 (11.9) | 0 (0) |
| **2016** | 1,841 (12.3) | 0 (0) | 1,694 (12.2) | 0 (0) | 1,334 (11.6) | 0 (0) |
| **2017** | 2,096 (13.9) | 0 (0) | 1,911 (13.8) | 0 (0) | 1,567 (13.6) | 0 (0) |
| **2018** | 2,110 (14.0) | 0 (0) | 1,957 (14.2) | 0 (0) | 1,676 (14.6) | 0 (0) |
| **2019** | 2,148 (14.3) | 0 (0) | 2,034 (14.7) | 0 (0) | 1,824 (15.9) | 0 (0) |
| **2020** | 1,903 (12.7) | 0 (0) | 1,822 (13.2) | 0 (0) | 1,721 (15.0) | 0 (0) |
| **Number of mental disorders** | | | | | | |
| **0** | 2,408 (16.0) | 64,960 (86.8) | 2,210 (16.0) | 60,065 (87.1) | 1,946 (16.9) | 50,233 (87.4) |
| **≥1** | 12,620 (84.0) | 9,836 (13.2) | 11,619 (84.0) | 8,886 (12.9) | 9,559 (83.1) | 7,254 (12.6) |

*“Having a yearly medical record for ADHD” was added to the definition of the ADHD population.

******“Having yearly medical record for both ADHD and dispensing dates for ADHD medication” was added to the definition of the ADHD population.

ADHD, attention deficit hyperactivity disorder; SD, standard deviation.

**Supplementary Table 6.** Proportion of ADHD and control groups with somatic diseases in the main and sensitivity analysis.

|  |  | | | **Sensitivity analysis_1*** | | | **Sensitivity analysis_2**** | | |
| --- | --- | --- | --- | --- | --- | --- | --- | --- | --- |
| **Somatic diseases**  **n (%)** | **ADHD**  **N=15,028**  **n (%)** | **Control**  **N=74,796**  **n (%)** | **Odds ratio*****  **(95% CI)** | **ADHD**  **N=13,829**  **n (%)** | **Control**  **N=68,951**  **n (%)** | **Odds ratio*****  **(95% CI)** | **ADHD**  **N=11,505**  **n (%)** | **Control**  **N=57,487**  **n (%)** | **Odds ratio*****  **(95% CI)** |
| **T2DM** | 435 (2.9) | 1,072 (1.4) | **2.05 (1.83, 2.29)** | 416 (3.0) | 988 (1.4) | **2.13 (1.90, 2.40)** | 344 (3.0) | 835 (1.5) | **2.09 (1.84, 2.37)** |
| **Diabetes complications** | 155 (1.0) | 400 (0.5) | **1.94 (1.61, 2.34)** | 147 (1.1) | 373 (0.5) | **1.98 (1.63, 2.39)** | 122 (1.1) | 308 (0.5) | **1.99 (1.61, 2.46)** |
| **Hypertension** | 994 (6.6) | 2,891 (3.9) | **1.76 (1.64, 1.90)** | 959 (6.9) | 2,777 (4.0) | **1.78 (1.65, 1.92)** | 796 (6.9) | 2,182 (3.8) | **1.88 (1.73, 2.05)** |
| **CVD** | 448 (3.0) | 1,256 (1.7) | **1.80 (1.61, 2.01)** | 420 (3.0) | 1,181 (1.7) | **1.80 (1.61, 2.01)** | 336 (2.9) | 943 (1.6) | **1.80 (1.59, 2.05)** |
| **Dyslipidemia** | 1,104 (7.4) | 2,722 (3.6) | **2.10 (1.95, 2.26)** | 1,055 (7.6) | 2,606 (3.8) | **2.10 (1.95, 2.26)** | 859 (7.5) | 2,087 (3.6) | **2.14 (1.97, 2.33)** |
| **Gout and hyperuricemia** | 667 (4.4) | 1,936 (2.6) | **1.75 (1.60, 1.91)** | 631 (4.6) | 1,837 (2.7) | **1.75 (1.59, 1.92)** | 508 (4.4) | 1,486 (2.6) | **1.74 (1.57, 1.93)** |
| **COPD** | 251 (1.7) | 576 (0.8) | **2.19 (1.89, 2.54)** | 234 (1.7) | 522 (0.8) | **2.26 (1.93, 2.64)** | 189 (1.6) | 427 (0.7) | **2.23 (1.88, 2.65)** |
| **NAFLD/NASH** | 619 (4.1) | 1,341 (1.8) | **2.35 (2.14, 2.59)** | 583 (4.2) | 1,273 (1.9) | **2.34 (2.12, 2.59)** | 474 (4.1) | 1,013 (1.8) | **2.40 (2.14, 2.68)** |
| **Atopic dermatitis** | 1,331 (8.9) | 4,432 (5.9) | **1.54 (1.45, 1.64)** | 1,213 (8.8) | 4,065 (5.9) | **1.53 (1.44, 1.64)** | 998 (8.7) | 3,418 (6.0) | **1.50 (1.40, 1.62)** |

*“Having a yearly medical record for ADHD” was added to the definition of the ADHD population.

******“Having yearly medical record for both ADHD and dispensing dates for ADHD medication” was added to the definition of the ADHD population.

*******Odds ratio for the ADHD/control group in the matched analysis set.

ADHD, attention deficit hyperactivity disorder; CI, confidence interval; COPD, chronic obstructive pulmonary disease; CVD, cardiovascular disease; NAFLD, non-alcoholic fatty liver disease; NASH, non-alcoholic steatohepatitis; T2DM, type 2 diabetes mellitus.

# Supplementary figures

**Supplementary Figure 1.** Logistic regression analyses adjusted for the presence or absence of mental disorders, the presence or absence of mental disorders-age, and the presence or absence of mental disorders-sex (the last two were interaction terms) in the ADHD group for all somatic diseases of interest (matched analysis set).


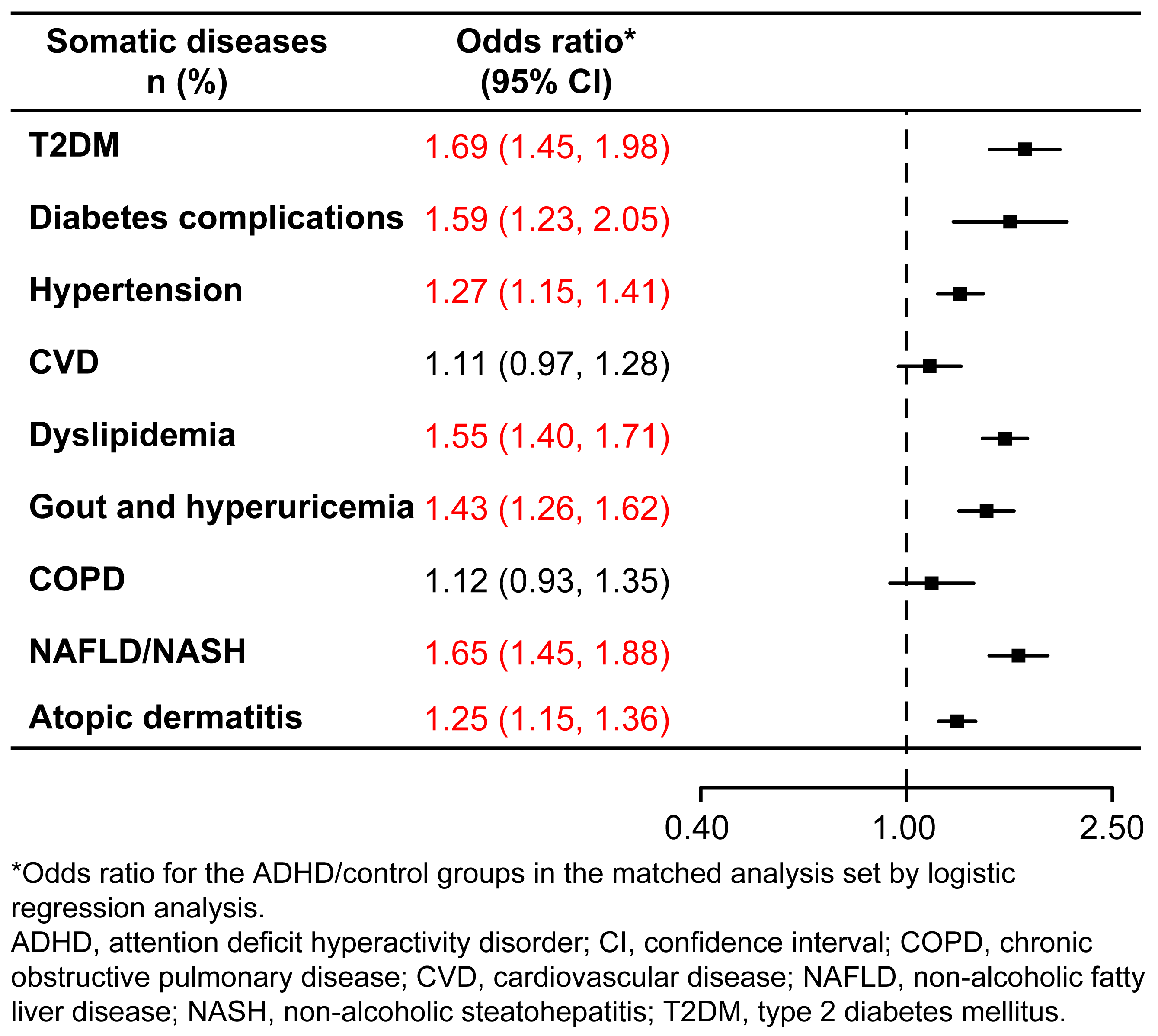


**Supplementary Figure 2.** Somatic diseases stratified by the presence or absence of mental disorders in the 18–29 years age category (matched analysis set).


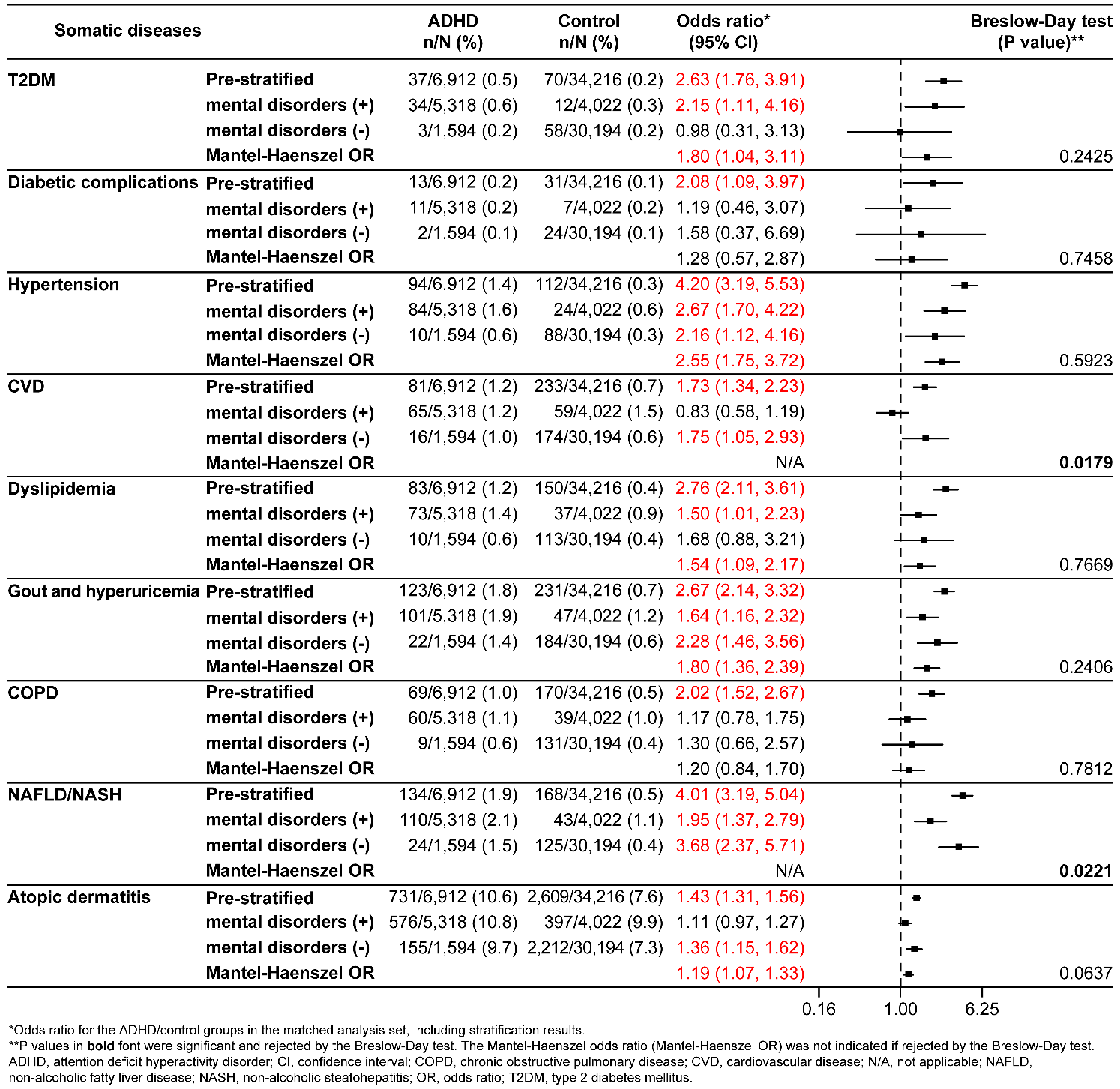


**Supplementary Figure 3.** Somatic diseases stratified by the presence or absence of mental disorders in the 30–39 years age category (matched analysis set).


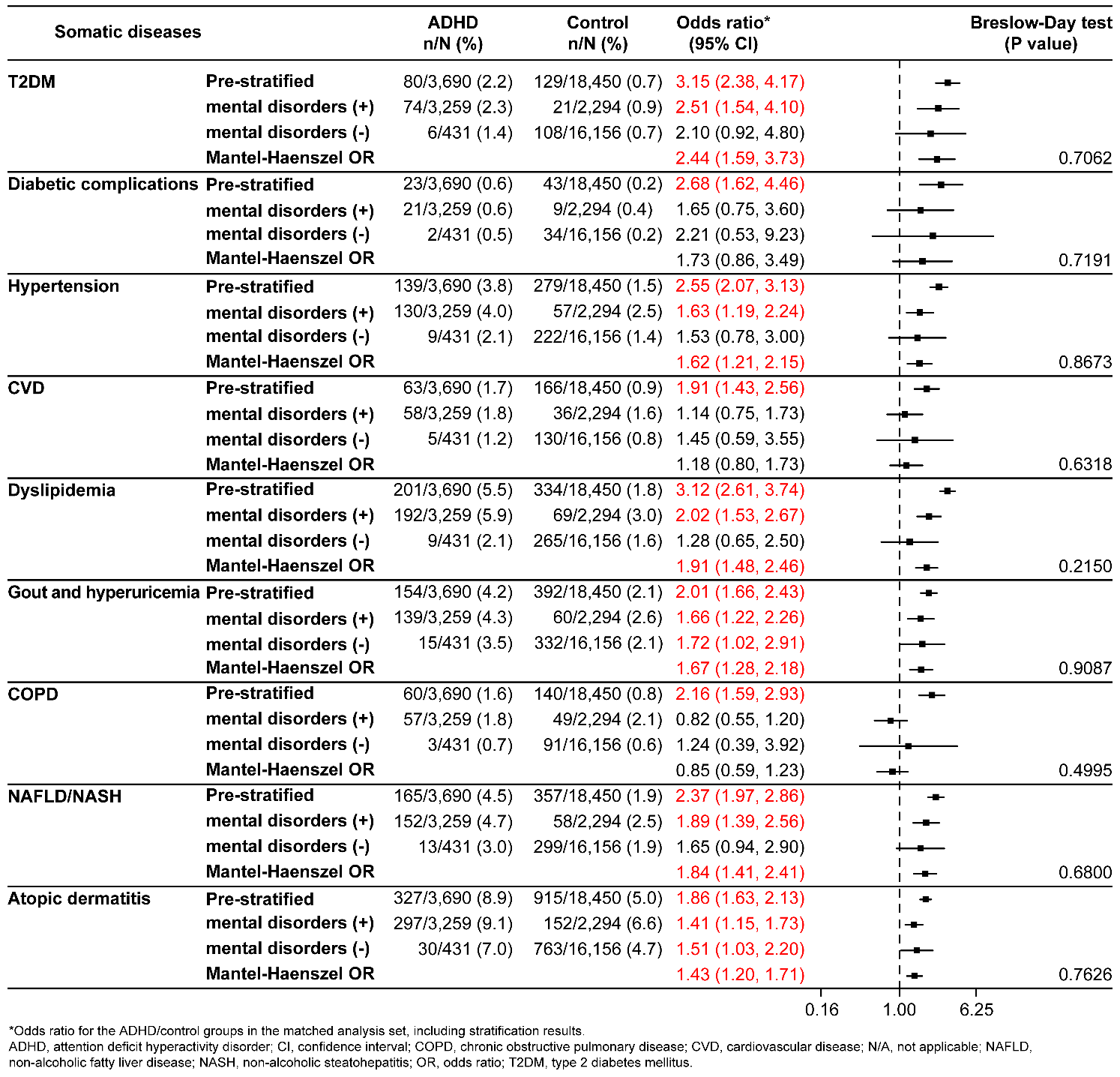


**Supplementary Figure 4.** Somatic diseases stratified by the presence or absence of mental disorders in patients with ADHD with yearly medical records (sensitivity analysis, matched analysis set).


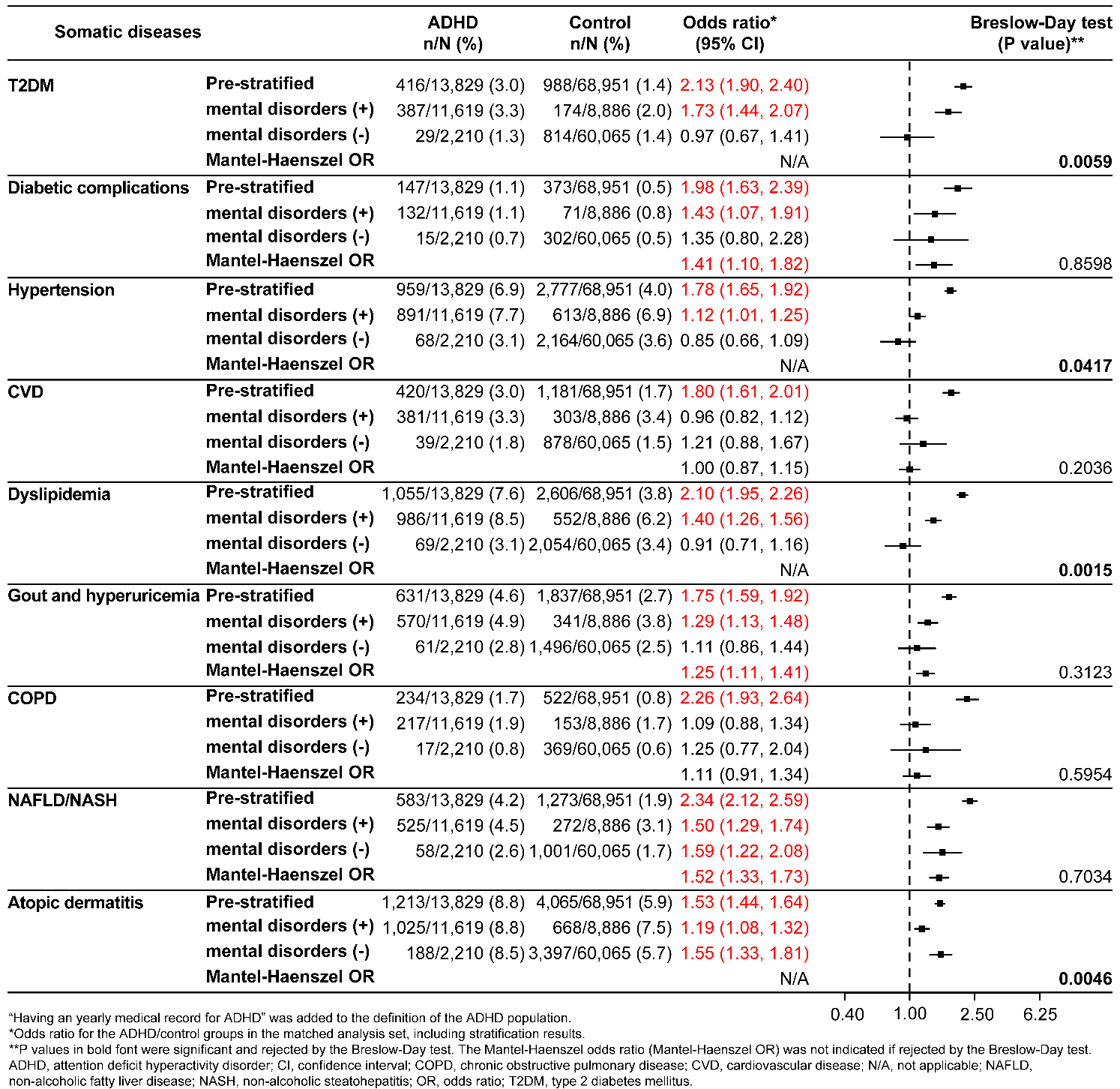


**Supplementary Figure 5.** Somatic diseases stratified by the presence or absence of mental disorders in patients with ADHD with yearly medical records and dispensing dates for ADHD medication (sensitivity analysis, matched analysis set).


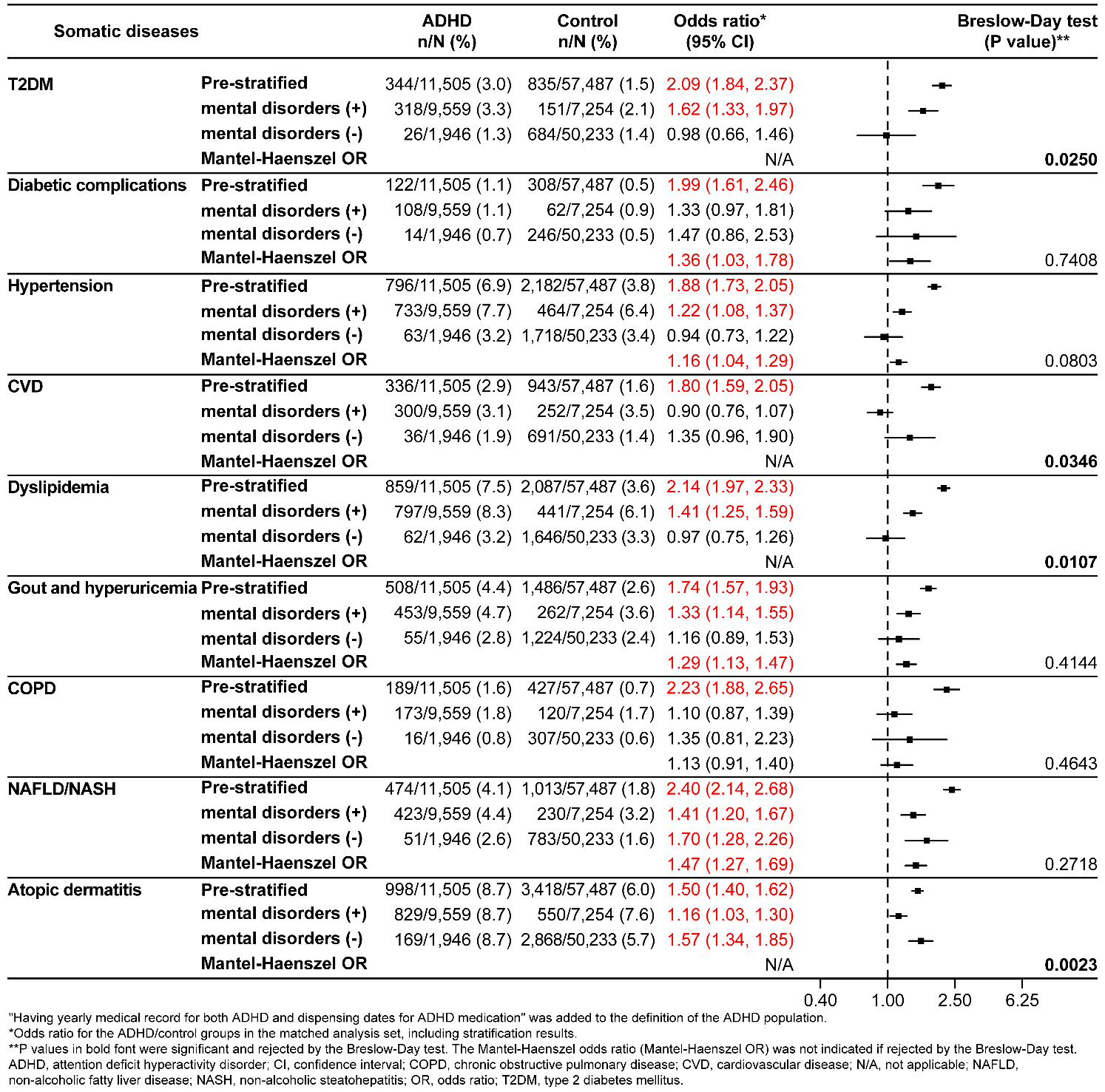


# Supplementary references

1. Gouda M, Matsukawa M, Iijima H. Associations between eating habits and glycemic control and obesity in Japanese workers with type 2 diabetes mellitus. *Diabetes Metab Syndr Obes* (2018) 11:647–58. doi: 10.2147/DMSO.S176749
2. Yamashita S, Imai S, Momo K, Kashiwagi H, Sato Y, Sugawara M, et al. Investigation of the real-world situation and risk factors associated with olanzapine prescribed to diabetes patients by using a Japanese claims database. *Biol Pharm Bull* (2021) 44:1151–5. doi: 10.1248/bpb.b21-00170
3. Hara A, Hirata T, Okamura T, Kimura S, Urushihara H. Lifestyle behaviors associated with the initiation of renal replacement therapy in Japanese patients with chronic kidney disease: a retrospective cohort study using a claims database linked with specific health checkup results. *Environ Health Prev Med* (2021) 26:102. doi: 10.1186/s12199-021-01022-3
4. Tanabe H, Saito H, Kudo A, Machii N, Hirai H, Maimaituxun G, et al. Factors associated with risk of diabetic complications in novel cluster-based diabetes subgroups: a Japanese retrospective cohort study. *J Clin Med* (2020) 9:2083. doi: 10.3390/jcm9072083
5. Ohishi M, Yoshida T, Oh A, Hiroi S, Takeshima T, Otsuka Y, et al. Analysis of antihypertensive treatment using real-world Japanese data-the retrospective study of antihypertensives for lowering blood pressure (REAL) study. *Hypertens Res* (2019) 42:1057–67. doi: 10.1038/s41440-019-0238-2
6. Kaneko H, Itoh H, Kiriyama H, Kamon T, Fujiu K, Morita K, et al. Restfulness from sleep and subsequent cardiovascular disease in the general population. *Sci Rep* (2020) 10:19674. doi: 10.1038/s41598-020-76669-z
7. Wake M, Onishi Y, Guelfucci F, Oh A, Hiroi S, Shimasaki Y, et al. Treatment patterns in hyperlipidaemia patients based on administrative claim databases in Japan. *Atherosclerosis* (2018) 272:145–52. doi: 10.1016/j.atherosclerosis.2018.03.023
8. Koto R, Nakajima A, Horiuchi H, Yamanaka H. Real-world treatment of gout and asymptomatic hyperuricemia: a cross-sectional study of Japanese health insurance claims data. *Mod Rheumatol* (2021) 31:261–9. doi: 10.1080/14397595.2020.1784556
9. Konomura k, Nagai H, Akazawa M. Development of a chronic obstructive pulmonary disease severity classification system using a Japanese health insurance claims database *Journal of Aging Research and Healthcare* (2017) 2:1–12. doi: 10.14302/issn.2474-7785.jarh-17-1727
10. Hasegawa K, Aruga A. Real-world data analysis of healthcare resource and cost utilization from non-alcoholic fatty liver disease patients in Japan. Research square [Preprint] (2021). doi: 10.21203/rs.3.rs-201639/v1. Available from: <https://www.researchsquare.com/article/rs-201639/v1> [Accessed March 20, 2023]
11. Kamei K, Saeki H, Tsuchiya T, Hirose T, Campos-Alberto E, Matsumoto F, et al. Real-world treatment patterns of patients with atopic dermatitis in Japan: analysis of the JMDC Claims Database. *J Cutan Immunol Allergy* (2021) 4:109–19. doi: 10.1002/cia2.12180
